# Sensor-Based Quantitative Assessment of Children’s Fine Motor Competence: An Instrumented Version of the Placing Bricks Test

**DOI:** 10.3390/s24072192

**Published:** 2024-03-29

**Authors:** Maria Cristina Bisi, Rita Stagni

**Affiliations:** 1 Department of Electrical, Electronic and Information Engineering “Guglielmo Marconi”, University of Bologna, Via del Risorgimento 2, 40136 Bologna, Italy; rita.stagni@unibo.it; 2 Interdepartmental Center for Industrial Research on Health Sciences & Technologies, University of Bologna, 40064 Bologna, Italy

**Keywords:** inertial sensors, fine motor competence, motor development

## Abstract

The assessment of fine motor competence plays a pivotal role in neuropsychological examinations for the identification of developmental deficits. Several tests have been proposed for the characterization of fine motor competence, with evaluation metrics primarily based on qualitative observation, limiting quantitative assessment to measures such as test durations. The Placing Bricks (PB) test evaluates fine motor competence across the lifespan, relying on the measurement of time to completion. The present study aims at instrumenting the PB test using wearable inertial sensors to complement PB standard assessment with reliable and objective process-oriented measures of performance. Fifty-four primary school children (27 6-year-olds and 27 7-year-olds) performed the PB according to standard protocol with their dominant and non-dominant hands, while wearing two tri-axial inertial sensors, one per wrist. An ad hoc algorithm based on the analysis of forearm angular velocity data was developed to automatically identify task events, and to quantify phases and their variability. The algorithm performance was tested against video recordings in data from five children. Cycle and Placing durations showed a strong agreement between IMU- and Video-derived measurements, with a mean difference <0.1 s, 95% confidence intervals <50% median phase duration, and very high positive correlation (ρ > 0.9). Analyzing the whole population, significant differences were found for age, as follows: six-year-olds exhibited longer cycle durations and higher variability, indicating a stage of development and potential differences in hand dominance; seven-year-olds demonstrated quicker and less variable performance, aligning with the expected maturation and the refined motor control associated with dominant hand training during the first year of school. The proposed sensor-based approach allowed the quantitative assessment of fine motor competence in children, providing a portable and rapid tool for monitoring developmental progress.

## 1. Introduction

Fine motor competence is usually defined as the ability of an individual to make precise, voluntary, and coordinated movements with their hands [1], and is considered a fundamental domain of motor control [2]. When this ability is compromised, fine motor difficulties emerge, possibly impacting individuals across all age groups, but clearly holding particular significance during children’s developmental stages. Children with fine motor development problems have difficulties with learning fine motor skills. They experience, for instance, problems with school tasks such as writing or cutting, or daily life activities such as closing a zipper or tying shoelaces [3]. Fine motor competence has been found to independently predict social and cognitive ability in pre-kindergarten children [1], emphasizing the interconnected development of problem-solving skills with the physical manipulation of the environment, and the role of fine motor skills in social play. Moreover, it has been shown that developing object manipulation skills in childhood promotes future physical activity [4].

Today, 5–10% of children in elementary school have developmental motor problems [3], thus, monitoring children’s fine motor development is fundamental to investigate underlying neurological disorders and design early effective interventions that can possibly mitigate the impact of motor developmental problems. 

Several tests have been proposed for the characterization of fine motor control, often being included in standardized assessment methods for measuring motor function (both gross and fine) [5]. Grip force scaling, speed of movement, and motor coordination are considered the three major components of fine motor control, and thus, they are target aspects to monitor [1]. While the choice of the task to be assessed usually takes into account these components, the metrics of evaluation are mainly based on qualitative observation, limiting quantitative assessment to the measure of test durations and/or number of errors (e.g., fine MABC-2, [6]). 

Recently, the integration of motion devices in observational methods was suggested to provide a quantitative and reliable assessment of motor skills for analyzing children’s motor competence [7].

Inertial wearable sensors allow to unobtrusively record and quantify movement for instrumented testing, complementing the information derived from qualitative observations with the quantification of significant parameters [8]. In recent years, inertial sensors have proven their effectiveness for motor assessment in elderly and/or pathological adult populations [8], and for the assessment of gross motor development in children [9,10].

With respect to fine motor competence, previous technology-based approaches were proposed using writing tablets, pressure sensitive drawing/writing utensils, and with lab-based motion capture systems [10]. When aiming to analyze the process of how a movement is performed, motion capture systems are preferrable. Recently, Niechwiej-Szwedo et al., [11] confirmed the feasibility and the promising pathway of performing a quantitative kinematic assessment within an optometric setting using inexpensive, portable, off-the-shelf equipment (Leap motion capture system) for enhancing the information provided by a routine motor function screening test (bead-threading task, fine MABC-2, [6]). They proposed a quantitative method for estimating the duration of sub-phases of each bead-threading trial (reach to bead, grasp and pick up bead, reach to needle, and place the bead on a needle), based on the analysis of hand velocity trajectory. By comparing results of two children with amblyopia and typically developing children, the study confirmed the advantages of assessing quantitatively the duration of the different task phases. Such analysis offers valuable insights into which aspects of the task present difficulties for the child, aiding in the diagnosis and management of treatment outcomes.

As an alternative to optometric settings, wearable sensors offer well-known advantages such as portability, low cost, and ease of use [12]. Moreover, for applications requiring widespread adoption, the presence of Inertial Measurement Unit (IMU) technology in commercial smartwatches provides a distinct advantage. To the authors’ knowledge no study has investigated the usability of inertial sensors for the quantitative assessment of fine motor competence.

Among the many available tests, the Placing Bricks (PB) test, part of the Test of Motor Competence (TMC) [2], was proposed to assess fine motor competences from infancy to old age. The TMC (including gross and fine motor performance assessment) was developed with the aim of defining an approach that (i) is sensitive at both ends of the scoring scale, (ii) minimizes ceiling effects, (iii) includes test items that can be performed by both very young children and very old people, and (iv) is easy to administer and does not require specialized training for experimenters.

The TMC was found to be applicable for a wide age-span (5–83 years) and favorable for longitudinal monitoring of fine and gross motor competence throughout the whole life-course. These characteristics answer to some of the latest major challenges highlighted in the literature regarding the study of motor development [13] (i.e., the lack of life span measures of motor competence, the lack of assessment feasibility for conducting research with large samples, and the limited sensitivity and discriminatory capabilities of assessments).

In PB standard assessment, participants are requested to attach eighteen square-shaped (2 × 2) Duplo™ bricks on a board (3 × 6 bricks size) as fast as possible [2]; PB performance measure is time to completion in seconds [2]. The instrumentation of the PB test using wearable inertial sensors can provide a more detailed, reliable, and quantitative characterization of the task, and thus of fine motor performance, enriching its informative power as referred to speed of movement and motor coordination, and overcoming the limitation of an analysis limited to product measurement. As it involves the repetition of a movement (grasping and placing), this test is well-suited for instrument-based assessment of the duration of its phases (e.g., brick placing time) and their variability across repetitions. These aspects are indeed linked to the performance and maturation of movement control [14].

The present study aims at instrumenting the PB test using wearable inertial sensors to complement PB standard assessment (time to completion) with reliable and objective process-oriented measures of performance. Quantitative data and ad hoc developed and tested algorithms can provide further insights into how the test is performed. In particular, the approach presented in this work exploits time-based metrics, extracted from sensor data, describing temporal phases’ duration and their variability.

In order to demonstrate the exploitability of the proposed approach, the present study did the following: (1) designed and tested a method capable of extracting time-based metrics (phase duration and phase duration variability) from wearable inertial sensors positioned on the wrist; (2) applied this method to analyze the influence of different factors (i.e., age, sex, hand dominance) on fine motor competence in a reference population of children.

## 2. Materials and Methods

### 2.1. Study Subjects

Fifty-four participants (27 females and 27 males) were included in the study (Table 1). They were divided into two age groups based on the attended school year (I grade 6-year-old children, 6YC, and II grade, 7-year-old children, 7YC). All children were born at full term (born at >36 weeks gestational age [15]) and had no known developmental delay and no musculoskeletal pathology. Children were excluded from the study if they had any severe visual or hearing impairment, used aids (except for glasses), had cochlear implants, or in case of a lack of cooperation. Participants’ information is reported in Table 1.

The Bioethics Committee of the University of Bologna approved this study (date of approval, 25 May 2016), and informed consent was obtained from the participants’ parents.

### 2.2. Experimental Setup

Two tri-axial wireless inertial sensors (OPAL, APDM Wearable Technologies, Portland, OR, USA) were mounted on the right and left wrists using elastic belts. The sensing axes were oriented along the anatomical longitudinal (L-x), medio-lateral (ML-y), and antero-posterior direction (AP-z).

The assessment was performed in schools, in a well-lit and ventilated room with adequate heat and sound. The test was executed following TMC guidelines [2]: the children were assessed wearing comfortable clothes, while sitting on a school chair in front of a school desk; they were given a practice run before the actual testing; the bricks were positioned in horizontal rows of three on the side of the active hand, resting on the side of the board, and the board was held firmly with the other hand; at start, the child lifts the active hand and grabs one brick with the active hand, carries it to the board, places it, and moves back the hand to grab another brick; the cycle is repeated until completion, when all bricks, one at a time, are placed on the board, and the active hand is positioned at rest on the side of the board. Both hands were tested. Standard PB performance assessment (time to completion in seconds, PBtime) was recorded using a stopwatch.

**Figure 1:**
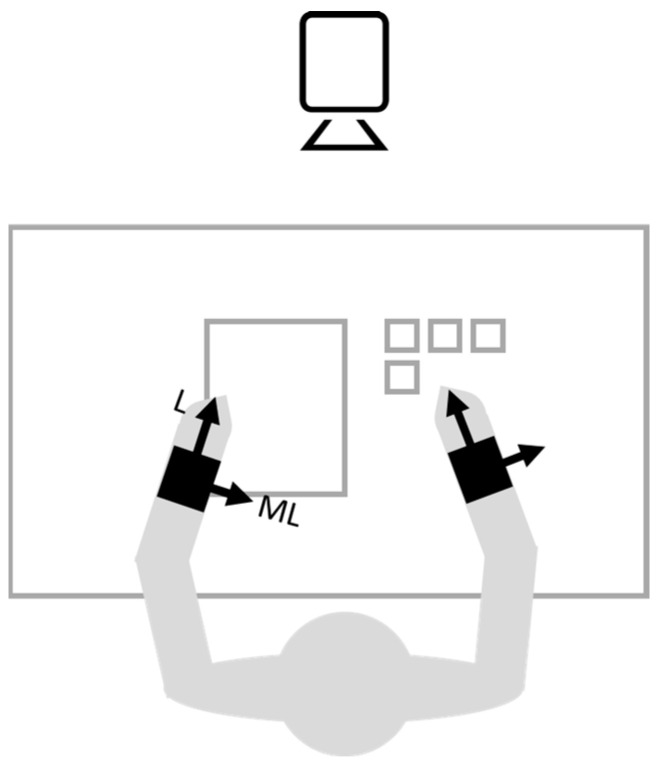
PB measurement setup. Black squares indicate sensors and arrows indicate axis orientation.

During actual testing, 3D forearm angular velocity (sampling frequency, 128 Hz) was measured. Tests were also filmed using a video camera (Hero4, GoPro, San Mateo, CA, USA, sampling frequency, 120 Hz, 848 × 480 pixels resolution) for reference. The video camera was positioned in front of the desk, capturing the whole desk within its field of view, along with the trunk and arms of the child. Figure 1 shows measurement setup and sensor positioning.

### 2.3. Data Analysis

**Figure 2:**
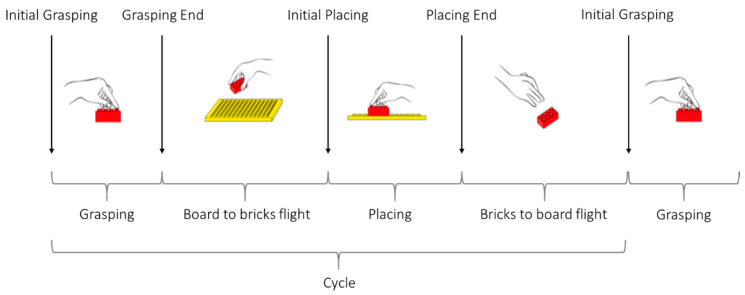
Placing Bricks phases (*Grasping*, Board to bricks flight, *Placing*, Bricks to board flight, and *Cycle*) and task events (Initial Grasping, Grasping End, Initial Placing, and Placing End).

The following 4 task events (TEs) were defined for phase segmentation: (i) Initial Grasping; (ii) Grasping End; (iii) Initial Placing; and (iv) Placing End. Task cycle duration (*Cycle*) was defined from a TE to the following same TE, and divided into the following 4 phases: (i) brick grasping (*Grasping*, from Initial Grasping to Grasping End); (ii) bricks to board flight (from Grasp End to Initial Placing); (iii) brick placing (*Placing*, from Initial Placing to Placing End); and (iv) board to bricks flight (from Placing End to Initial Grasping). Thus, the test consisted of 1 initial phase (i.e., from rest at start to the first Initial Grasping), 17 full Task cycles, and 1 final phase (i.e., from the final Placing End to rest at stop). An illustrative description of TEs and phases is shown in Figure 2.

**Figure 3:**
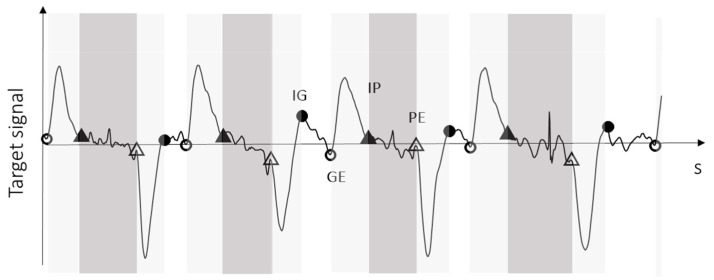
Exemplificative TEs identification on the target signal. Triangles represent Initial Placing (full triangles) and Placing End (white triangles). Circles represent Initial Grasping (black circles) and Grasping End (white circles). Flight phases are highlighted in light gray, *Placing* in dark gray, and *Grasping* in white.

An ad hoc algorithm was developed to identify TEs on 3D forearm angular velocity (TE_IMU_). To take into account different arm orientations (around L and AP axes) during the execution of the task and to retain the peak signs for feature identification, the algebraic sum of angular velocity components around the L and the AP axes was selected as the target variable. The target variable was low pass filtered with a 4th order Butterworth filter with a cut-off frequency of 6 Hz. Absolute peaks of the target variable were identified as associated to flight phases (discriminating the direction of brick to board flight phase, positive, and board to brick phase, negative), then, minima before and after each flight peak were identified as associated to brick *Grasping* and brick *Placing*, respectively. Figure 3 shows an exemplificative target signal with TE_IMU_ identification.

#### 2.3.1. Algorithm Test

TEs were (i) visually identified from the video recordings (TE_GoPro_) by one operator and (ii) extracted from the collected data using the algorithm described above (TE_IMU_), from video and IMU data of 5 children (2 6YC and 3 7YC, 2F/3M; age: median (min–max), 90 (77–95) months; height: 1.28 (1.08–1.29) m; and body mass: 25.0 (21.0–30.7) kg) for both hands, for a total of 10 tests and 180 Initial Grasping, Grasping End, Initial Placing, and Placing End. TEs_GoPro_ were considered as reference.

Additionally, to quantify inter-rater variability, three operators independently conducted visual assessments on the tests of a single participant (both hands).

##### Statistical Analysis

The sensitivity and the positive predictive value (PPV) in TE identification were calculated [16], respectively, as

(1) $$Sensitivity=100\times \frac{Number\;of\;TEs\;correctly\;identified\;by\;algorithm}{Number\;of\;all\;TEs\;as\;identified\;from\;video}$$

(2) $$PPV=100\times \frac{Number\;of\;TEs\;correctly\;identified\;by\;algorithm}{Number\;of\;all\;TEs\;identified\;by\;algorithm}$$

TEs_IMU_ were deemed to be correctly identified if, on the video, they corresponded to the second half of the phase immediately preceding the TE under consideration, or to the first half of the phase immediately following it.

TE showing the highest Sensitivity and PPV results was selected for *Cycle* calculation (time duration from one TE to the following TE of the same type).

The phase durations in seconds of the 17 full task cycles (*Grasping*, brick to board flight, *Placing*, board to brick flight, and *Cycle*) were calculated from the identified TEs.

The maximum inter-rater mean difference of phase duration and the widest 95% confidence interval of the mean were extracted as benchmarks for visual assessment.

Phase duration derived from TE_GoPro_ and from TE_IMU_ were compared using Bland Altman plots [17] and Pearson’s correlation coefficients (ρ). Phase durations estimated from IMU data were considered reliable when

- Bland Altman plots showed mean differences of <0.1 s and a 95% confidence interval <50% of the median phase duration;
- Pearson’s correlation resulted very strong (ρ > 0.9).

#### 2.3.2. Analysis of the Effect of Age, Sex, and Hand Dominance

Data from all participants were analyzed. Reliable phases were extracted and expressed in percentage of the corresponding *Cycle*. Median, interquartile (IQR), and Range of each phase were calculated for each participant. Short- and long-term variability of temporal phases were calculated using Poicaré plots (SD1, short-term, SD2, long-term variability) [18].

##### Statistical Analysis

Normal distributions of PB standard assessment (PBtime) and of IMU-based measures (phase durations and phase variability) on the different groups (i.e., males, females, 6YC, 7YC, dominant hand, non-dominant hand) and on the entire dataset was tested using a Kolmogorov–Smirnoff test; normal distribution was not verified for all of the parameters.

A Mann–Whitney U test was applied to PBtime and to IMU-based measures (significance level 0.05) to test differences between

- male and female participants;
- 6YC and 7YC.

A Wilcoxon signed rank test for repeated measures was applied (significance level 0.05) to test differences between the dominant and non-dominant hand.

Data and statistical analyses were performed in MATLAB2023a (MathWorks BV, Natick, MA, USA).

## 3. Results

### 3.1. Algorithm Test

From TE analysis, sensitivity results ranged from 90.59% for Initial Grasping to 97.65% for Initial Placing. PPVs ranged from 94.71% for Grasping End to 97.65% for Initial Placing and Placing End. Table 2 shows sensitivity and PPV results for each of the TEs.

Initial Placing resulted the TE with the highest Sensitivity and PPV, thus, it was chosen for *Cycle* calculation. *Cycle* was then defined as the time duration from an Initial Placing to the following Initial Placing.

Inter-rater mean difference, assessed on the videos of one participant, resulted lower than 0.09 s for all of the considered phases, and the 95% confidence interval ranged from 0.11 s for *Placing* to 0.23 s for *Grasping* (Table 3).

**Figure 4:**
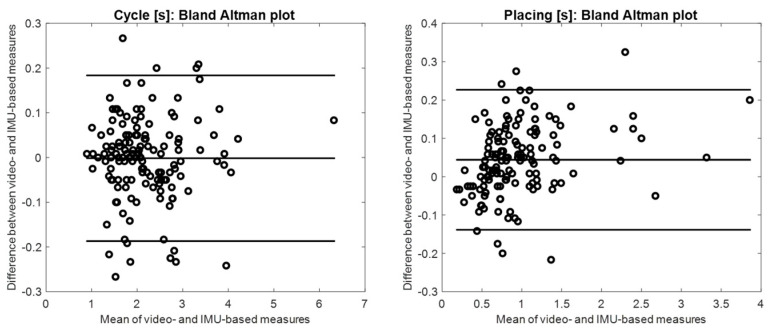
Bland Altman plots of *Cycle* [s] and *Placing* [s] obtained from video recordings and IMU data (mean and 95% confidence interval, solid lines).

The mean differences between Video- and IMU-based estimation resulted lower than 0.07 s for all of the considered phases, and the 95% confidence interval ranged from 0.19 s for *Placing* and *Cycle* duration to 0.35 s for *Grasping* and board to bricks flight duration (Table 4). Bland Altman plots for *Cycle* and *Placing* are shown in Figure 4.

The following significant positive correlations were found for all of the analyzed phases: weak to moderate correlations (0.1 < ρ < 0.6) for flight phases, strong correlation (0.6 < ρ < 0.9) for *Grasping*, and very strong correlations (ρ > 0.9) for *Placing* and *Cycle* phases. Table 4 shows mean differences, 95% confidence intervals, and Pearson’s correlation coefficients for each phase.

*Cycle* and *Placing* resulted reliable (mean difference < 0.1 s, 95% confidence interval <50% of median phase duration, and ρ > 0.9) and were considered for further analysis. *Grasping* and flight phases showed a 95% confidence interval ranging from 93% (Bricks to board flight) to 318% (for *Grasping*) of their median duration, and Pearson’s correlation coefficients lower than 0.9.

### 3.2. Effect of Age, Sex, and Hand Dominance

No significant differences were found between male and female participants for standard assessment (PBtime) and for the estimated temporal parameters (*Cycle*, *Placing*, and their variability).

When performing the task with the dominant hand, 7YC showed shorter median values of PBtime, as well as *Cycle* and *Placing* durations, than 6YC. For the dominant hand, 7YC also showed significantly lower variability of *Cycle* (IQR, Range, SD1, and SD2) than 6YC. Variability of *Placing* resulted lower too, but resulting differences were not significant. With the non-dominant hand, 7YC showed lower PBtime and *Cycle* duration than 6YC; IQR, Range, and SD1 of *Cycle* resulted lower too, while no statistical differences were found for *Placing* between the two age groups.

When comparing dominant and non-dominant hands, the dominant hand showed median values of PBtime, as well as *Cycle* and *Placing* duration that was significantly lower than the non-dominant hand. The same significant trend was found for all of the variability parameters applied both on *Cycle* and *Placing* (IQR, Range, SD1, and SD2).

Table 5 shows the 25th, 50th, and 75th percentiles of PBtime, and Table 6 shows the median duration and variability results of *Cycle* and *Placing*, divided by age groups and by dominant and non-dominant hand.

## 4. Discussion

In this study, PB test was instrumented using wearable inertial measurement units to provide quantitative process-oriented assessment of fine motor competence in children. The proposed approach extracts time-based metrics (phase durations and variability) from inertial sensor data, allowing for a detailed analysis of task execution. An ad hoc algorithm based on the analysis of forearm angular velocity data was developed and tested against video recordings, demonstrating high sensitivity and positive predictive values in identifying task events.

For *Placing* and *Cycle*, there was a strong agreement between IMU- and Video-derived phase durations, with mean differences and 95% confidence intervals within pre-defined acceptable ranges (mean difference <0.1 s, 95% confidence interval <50% of the median phase duration), and very strong positive correlation (ρ > 0.9). For *Grasping* and flight phases, the agreement was lower: children showed different strategies for *Grasping* (e.g., early stopping with the forearm and using only the hand, or, on the contrary, using no wrist strategy), influencing the accuracy of Initial Grasping and Grasping End identification, thus, the phases both preceding and succeeding these TEs. Furthermore, the median duration of *Grasping* and flight phase resulted shorter than *Placing* and *Cycle*, meaning that estimation errors had a greater impact on the results.

With respect to algorithm testing, it is also important to acknowledge that, while video recording for observational analysis is commonly used as a reference standard in motor performance assessments [7], it is not without limitations as it is inherently subjective, introducing human variability in event detection. Inter-rater mean difference assessed in the present work resulted comparable to the ones obtained between Video- and IMU-based phase measurements. The 95% confidence interval for inter-rater differences was slightly wider, but following the same trend (lowest values for *Cycle* and *Placing*). It is worth noting that these findings are based on assessments of only one child, whereas comparisons between Video- and IMU-based measurements were conducted on the tests of five children.

When examining the Bland–Altman plot for *Cycle* duration, shown in Figure 4, a slightly higher than expected number of outliers can be noticed (7%), indicating a potential deviation from a normal distribution in the mean difference of the measure. Upon reviewing the video frames corresponding to these *Cycles*, we observed that, in these cases, the children frequently exhibited discontinuous movements before or after the flight phase (e.g., a Bricks to Board flight phase followed by another small flight phase to adjust the placement position). These irregularities introduced greater errors in identifying the Initial Placing and, consequently, in estimating the *Cycle* duration. Indeed, three positive and three negative outliers resulted coupled in consecutive cycles (in three different children), supporting this observation.

In the second part of the study, the fine motor competence of 54 primary school children (27 I graders and 27 II graders) was quantified using the proposed method and analyzed for the effect of sex, age, and hand dominance.

Overall, the findings confirm anticipated differences in fine motor competence within the examined groups. While no distinctions based on sex were anticipated, in accordance to previous research findings [19], older age and the utilization of the dominant hand appear to facilitate more effective motor control and coordination, consistent with the recognized trajectory of fine motor development throughout childhood [20,21].

When analyzing differences between 6YC and 7YC groups, corresponding to two different school grades, results indicate that 7YC generally exhibited shorter task completion times (PBtime), *Cycle* and *Placing* durations, and lower variability of *Cycle* compared to 6YC, highlighting the expected improvements in fine motor skills with age and with school education. While direct comparison is not possible due to there being different tasks and age groups under consideration, *Placing* duration tended to decrease with age maturation as for the quantitative assessment of fine MABC-2 [11]. Differences were higher for the dominant hand, probably as a consequence of the intensive training that happens during the first year of primary school with the learning of writing skills. The presence of significant expected differences between the two closely spaced age groups is promising for the future applicability of this approach across a broader range of age groups.

Additionally, the dominance of the hand used in task execution influenced performance metrics too, as expected, with the dominant hand showing faster and less variable performance than the non-dominant hand for both *Cycle* and *Placing*.

One limitation of our study is that we only explored sensor positioning on the wrist: it is possible that using a different sensor positioning (e.g., on the hand) might have led to different testing outcomes. However, this would likely complicate the administration of the test, making it less feasible and easy to implement, which could hinder its widespread adoption (considering the availability of many smartwatches containing IMU technology). Thus, if a more accurate but less user-friendly result is desired, different sensor positions could be explored in the future.

The present study solely focused on phase durations and their variability as quantitative measures of motor performance; other quantitative metrics can potentially contribute to the assessment of fine motor competence [10]. This study serves as a preliminary investigation aimed at testing our approach for task sub-phase detection, and demonstrates the significance of this quantitative information in discriminating levels of motor competence; moreover, sub-phase segmentation will serve for extracting additional measures. Future works will investigate the possible use of other quantitative metrics to integrate and enhance the analysis of the PB test for fine motor competence assessment.

Overall, the study demonstrates the feasibility and utility of using wearable sensors to objectively assess fine motor skills in children through the quantitative kinematic assessment of the PB test. By providing quantitative measures of motor performance, the proposed approach offers valuable insights into the developmental trajectories of fine motor skills, offering the possibility to quantify possible alterations with respect to reference population. Moreover, the selection of the PB test and the widespread accessibility of wrist-worn IMU technology make the proposed approach applicable for a wide age-span (5–83 years) and favorable for longitudinal monitoring of fine motor competence throughout the whole life-course, allowing for the creation of age reference bands and datasets, supporting data interpretation. Future research will explore fine motor development using longitudinal study designs.

## Figures and Tables

**Table 1:** Number of participants (n) and participants’ characteristics (median (min–max)) for the two age groups (6YC = 6-year-old children, 7YC = 7-year-old children).

	n (Male/Female)	Age (Months)	Height (m)	Body Mass (kg)	BMI
6YC	27 (12F/15M)	78 (73–85)	1.22 (1.08–1.30)	25.6 (19.5–38.0)	17.4 (14.8–22.8)
7YC	27 (15F/12M)	91 (86–106)	1.25 (1.12–1.37)	28.0 (19.7–41.0)	17.1 (14.7–21.8)

**Table 2:** Sensitivity and PPV results for each estimated TE.

	Initial Grasping	Grasping End	Initial Placing	Placing End
Sensitivity	90.59	94.71	97.65	97.06
PPV	95.29	94.71	97.65	97.65

**Table 3:** Median Video-based duration [s] (25th and 75th percentiles) and inter-rater mean difference (md); 95% confidence interval (95% CI) of Video-based phase measurements.

	*Grasping*	Bricks to Board Flight	*Placing*	Board to Bricks Flight	*Cycle*
Video-based duration [s]	0.25 (0.21–0.45)	0.45 (0.41–0.51)	0.74 (0.65–0.96)	0.37 (0.33–0.47)	1.95 (1.84–2.14)
md	0.09	−0.02	0.03	−0.01	0.00
95% CI	±0.23	±0.17	±0.11	±0.13	±0.13

**Table 4:** Median Video-based duration [s] (25th and 75th percentiles) and mean difference (md); 95% confidence interval (95% CI) and Pearson’s correlation coefficient (ρ) between Video- and IMU-based phase measurements.

	*Grasping*	Bricks to Board Flight	*Placing*	Board to Bricks Flight	*Cycle*
Video-based duration [s]	0.22 (0.13–0.38)	0.49 (0.40–0.58)	0.80 (0.60–1.10)	0.46 (0.35–0.59)	2.02 (1.62–2.67)
md	0.07	−0.06	0.04	−0.04	0.00
95% CI	±0.35	±0.23	±0.19	±0.35	±0.19
ρ	0.80	0.47	0.99	0.31	0.99

**Table 5:** 25th, 50th, and 75th percentiles of PBtime [s] of 6YC and 7YC divided by dominant and non-dominant hand. Asterisks indicate significant differences (** *p* < 0.05), (dominant hand, Dh, Non-Dominant hand, NDh).

	6YC						7YC						6YC vs. 7YC		Dh vs. NDh
	Dominant Hand			Non-Dominant Hand			Dominant Hand			Non-Dominant Hand					
	25th	50th	75th	25th	50th	75th	25th	50th	75th	25th	50th	75th	Dh	NDh	
**PBtime (s)**	34.92	39.04	44.99	37.77	41.15	48.4	28.1	31.08	37.91	31.02	36.99	39.63	**	**	**

**Table 6:** 25th, 50th, and 75th percentiles of *Cycle* [s] and *Placing* (%*Cycle*) results (Median, IQR, Range, SD1, and SD2) of 6YC and 7YC divided by dominant and non-dominant hand. Asterisks indicate significant differences (** *p* < 0.05, * *p* < 0.1), (dominant hand, Dh, Non-Dominant hand, NDh).

		6YC						7YC						6YC vs. 7YC		Dh vs. NDh
		Dominant Hand			Non-Dominant Hand			Dominant Hand			Non-Dominant Hand					
		25th	50th	75th	25th	50th	75th	25th	50th	75th	25th	50th	75th	Dh	NDh	
***Cycle* (s)**	Median	1.80	2.08	2.28	2.02	2.21	2.68	1.43	1.68	2.03	1.62	1.91	2.12	**	**	**
	IQR	0.47	0.57	0.76	0.54	0.89	1.11	0.33	0.49	0.70	0.36	0.67	0.80	*	**	**
	Range	1.64	2.70	3.82	2.09	2.66	3.92	1.06	1.71	2.68	1.92	2.23	2.89	**	*	**
	SD1	0.49	0.67	1.05	0.57	0.77	0.93	0.30	0.47	0.67	0.50	0.62	0.73	**	*	**
	SD2	0.42	0.59	0.94	0.53	0.72	1.03	0.30	0.46	0.65	0.47	0.62	0.80	**		**
***Placing* (%*Cycle***)	Median	37.6	41.7	47.0	43.4	48.5	52.3	35.5	38.2	40.5	40.6	44.2	50.7	**		**
	IQR	13.2	17.8	21.5	14.3	25.8	42.5	11.1	13.6	20.1	11.5	16.5	36.2			**
	Range	42.3	57.9	85.8	47.4	80.5	89.8	31.6	46.5	66.5	48.6	80.9	91.4			**
	SD1	9.9	13.8	20.3	13.1	22.3	30.0	7.8	13.0	16.9	12.7	21.6	27.8			**
	SD2	12.6	16.6	22.4	15.0	23.7	29.4	9.0	12.8	20.5	13.4	22.2	29.5			**

## Data Availability

The raw data supporting the conclusions of this article will be made available by the authors on request.
